# Safety of topical administration of fluralaner plus moxidectin concurrently with praziquantel in cats

**DOI:** 10.1186/s13071-018-3170-4

**Published:** 2018-11-19

**Authors:** Feli M. Walther, Petr Fisara, Rainer K. A. Roepke

**Affiliations:** 1 Merck Animal Health, 2 Giralda Farms, Madison, NJ 07940 USA; 2 MSD Animal Health, 26 Artisan Road, Seven Hills, NSW Australia; 3 MSD Animal Health Innovation GmbH, Zur Propstei, Schwabenheim, Germany

**Keywords:** Cat, Fluralaner, Moxidectin, Bravecto™ Plus, Praziquantel, Droncit™, Safety

## Abstract

**Background:**

Fluralaner provides efficacy against feline ectoparasites following topical administration. Moxidectin is routinely used to treat gastrointestinal nematode infections and prevent heartworm disease caused by *Dirofilaria immitis*. Praziquantel is routinely used to treat feline tapeworm infections. The safety of a fluralaner plus moxidectin combination topical solution (Bravecto™ Plus, MSD Animal Health) was assessed when administered concurrently with a commercially available praziquantel topical solution (Droncit™ Spot-on, Bayer Animal Health GmbH). The highest dose rates in clinical use were tested.

**Results:**

Concurrent topical administration of a fluralaner plus moxidectin and a praziquantel product did not result in adverse findings. One out of ten cats receiving praziquantel only (control group), and two out of ten cats receiving fluralaner plus moxidectin and praziquantel (treatment group) had dandruff-like flakes in their coat at the application site. Two out of the ten control cats and three cats out of the ten treatment group cats had very small amounts of unidentified material (minute crusts or crumbs) at the application site which was only visible during close inspection.

**Conclusions:**

The concurrent treatment of cats with fluralaner plus moxidectin and praziquantel at the maximum dose in clinical use was well tolerated.

## Background

Fluralaner is active against ectoparasite infestations in cats. Following a single topical application, fluralaner provides immediate and persistent flea (*Ctenocephalides felis*) and tick (*Ixodes ricinus*) killing activity for 12 weeks [1]. Moxidectin is active against gastrointestinal nematodes (fourth-stage larvae, immature adults and adults of *Toxocara cati* and *Ancylostoma tubaeforma*) and prevents heartworm disease caused by *Dirofilaria immitis* [1]. A combination of fluralaner plus moxidectin as a topical solution has been shown to be equally effective as a fluralaner-only product against ectoparasites [2, 3] whilst providing an effective treatment against gastrointestinal nematodes [4]. This combination has also been shown to be safe in healthy cats when administered topically at doses of up to 5 times the maximum clinical dose of 93 mg fluralaner and 4.65 mg moxidectin, i.e. at up to 465 mg fluralaner and 23.25 mg moxidectin/kg body weight, on three occasions 8 weeks apart [1].

Praziquantel is active against tapeworm in cats (*Dipylidium caninum*, *Taenia* spp., *Echinococcus multilocularis*) [5].

Cats may concurrently be infested with ectoparasites such as ticks or fleas as well as gastrointestinal helminths. Therefore, veterinarians may choose to administer an anticestodal agent such as praziquantel concurrently with fluralaner plus moxidectin topical solution. Fluralaner plus moxidectin (Bravecto™ Plus; MSD Animal Health, Boxmeer, The Netherlands) and praziquantel (Droncit™ Spot-on; Bayer Animal Health GmbH, Leverkusen, Germany) are topical formulations, providing efficacy after systemic absorption.

A previous study showed that fluralaner is well tolerated in cats when administered concurrently with an endectoparasiticide combination product containing emodepside and praziquantel [6]. The study presented below assessed the safety of topical administration of fluralaner plus moxidectin concurrently with praziquantel in healthy cats at the maximum dose rates in clinical use.

## Methods

Twenty healthy domestic breed cats (7 males and 13 females) were included in the study. This study was conducted in 2017 in Queensland, Australia, under the Australian Pesticides and Veterinary Medicines Authority (APVMA) General Permit PER7250 covering a defined range of Small Scale Trials.

Twenty cats were allocated to two study groups (control group and treatment group) using a randomized block method, ranked by sex then body weight (Table 1). Cats included in the study were 0.6–6.4 (mean 3.9) years of age and weighed 2.6–4.9 (mean 3.6) kg. Praziquantel was administered topically at a dose of 16.7 mg/kg body weight to cats of both control and treatment group on study day 0. Fluralaner plus moxidectin was administered to cats of the treatment group on study day 0 at the maximum treatment dose of 93 mg fluralaner + 4.65 mg moxidectin/kg body. Doses were calculated based on body weights determined on study day -1 and the calculated volume was applied topically at the base of the skull. For cats receiving both products, the fluralaner plus moxidectin solution was applied first at the base of the skull and the praziquantel solution was applied caudal thereto.

**Table 1:** Dosage and animal details of the control group and the treatment group included in the safety assessment of the concurrent use of fluralaner-moxidectin and praziquantel

		Control group	Treatment group
Fluralaner plus moxidectin treatment (study day)		–	0
Praziquantel treatment (study day)		0	0
Fluralaner dose (mg/kg body weight)		–	93
Moxidectin dose (mg/kg body weight)		–	4.65
Praziquantel dose (mg/kg body weight)		16.7	16.7
Sex	Male	3	4
	Female	7	6
Body weight pre-treatment (kg)	Mean ± SD	3.7 ± 0.7	3.5 ± 0.6
Body weight at study end (kg)	Mean ± SD	3.7 ± 0.7	3.6 ± 0.5

*Abbreviation*: *SD* standard deviation

Physical examinations were performed by a veterinarian on study days -7, -1, 14 and 28. Parameters assessed included appearance, body condition, musculoskeletal system and locomotion, palpable lymph nodes, skin and hair, abnormalities at the application site, palpation of the abdomen and pulse, auscultation of the heart, heart rate, auscultation of respiratory tract, respiratory rate, rectal temperature, sensory system including ears, eyes and pupils, oral cavity including mucous membranes and capillary refill time; any other abnormalities were recorded. General health observations were performed twice daily throughout the study and body weights were recorded weekly.

In addition, clinical assessments for abnormalities in behavior, locomotion, body orifices, pulse, eyes, mucous membranes, respiration, coat and skin (including application site) were carried out by a veterinarian for all study cats prior to treatment on day 0, during the first hour following treatment, and at 2, 4, 8, 12, 24, 36, 48 and 72 hours and at 4, 5, 6 and 7 days after treatment.

## Results and discussion

The concurrent application of fluralaner plus moxidectin and praziquantel (treatment group) did not result in findings considered to be related to the concurrent application of both products. All cats remained in good general health throughout the study.

One cat in the control group and two cats in the treatment group had dandruff-like flakes in their coat at the application site for up to three weeks after application. Two cats in the control group and three cats in the treatment group had very small amounts of unidentified material (minute crusts or crumbs) at the application site, which were only visible during close inspection of the application site, for up to two weeks after application. The skin was normal in all cases. One cat in the control group developed an over-grooming lesion within 12 hours of the praziquantel administration, which resolved by the end of the study. Findings at the application site occurred to a similar extent in control and treatment group cats, and are consistent with possible local reactions specified on the products’ labels [1, 5]. All cases were mild and none required concomitant medication.

Cases of mild epiphora and sniffling, and suspected overgrooming lesion at the abdomen were observed but are considered incidental and not related to the application of praziquantel and/or fluralaner-moxidectin. All cases were mild and did not require concomitant medication.

Body weights remained stable throughout the study in all cats (Table 1).

Whilst generated on a limited number of cats, the study results do not indicate a safety risk associated with the concurrent use of fluralaner-moxidectin and praziquantel at the maximum dose rates recommended for clinical use.

## Conclusions

Concurrent topical application of fluralaner plus moxidectin and praziquantel at the maximum recommended dose rates is well tolerated by cats.
